# *In Vitro* Fermentation of Selected Prebiotics and Their Effects on the Composition and Activity of the Adult Gut Microbiota

**DOI:** 10.3390/ijms19103097

**Published:** 2018-10-10

**Authors:** Sophie Fehlbaum, Kevin Prudence, Jasper Kieboom, Margreet Heerikhuisen, Tim van den Broek, Frank H. J. Schuren, Robert E. Steinert, Daniel Raederstorff

**Affiliations:** 1 DSM Nutritional Products Ltd., R&D Human Nutrition and Health, 4002 Basel, Switzerland; kwprudence1200r@gmail.com (K.P.); robert.steinert@dsm.com (R.E.S.); daniel.raederstorff@dsm.com (D.R.); 2 The Netherlands Organization for Applied Scientific Research (TNO), Microbiology and Systems Biology, 3704 HE Zeist, The Netherlands; jasper.kieboom@tno.nl (J.K.); margreet.heerikhuisen@tno.nl (M.H.); tim.vandenbroek@tno.nl (T.v.d.B.); frank.schuren@tno.nl (F.H.J.S.)

**Keywords:** XOS, beta-glucan, alpha-GOS, screening platform, i-screen, colonic fermentation

## Abstract

Recently, the concept of prebiotics has been revisited to expand beyond non-digestible oligosaccharides, and the requirements for selective stimulation were extended to include microbial groups other than, and additional to, bifidobacteria and lactobacilli. Here, the gut microbiota-modulating effects of well-known and novel prebiotics were studied. An *in vitro* fermentation screening platform (i-screen) was inoculated with adult fecal microbiota, exposed to different dietary fibers that had a range of concentrations (inulin, alpha-linked galacto-oligosaccharides (alpha-GOS), beta-linked GOS, xylo-oligosaccharides (XOS) from corn cobs and high-fiber sugar cane, and beta-glucan from oats), and compared to a positive fructo-oligosaccharide (FOS) control and a negative control (no fiber addition). All dietary fibers displayed prebiotic activity, with beta-glucan showing more distinct effects on the microbial composition and metabolism compared to the other fibers. Beta-glucan induced the growth of *Prevotella* and *Roseburia* with a concomitant increase in propionate production. Inulin and both forms of GOS and XOS had a strong bifidogenic effect on the microbial composition. A dose-response effect was observed for butyrate when exposed to beta-glucan and inulin. The findings of this study support the potential for alpha-GOS, XOS, and oat beta-glucan to serve as novel prebiotics, due to their association with the positive shifts in microbiome composition and short-chain fatty acid production that point to potential health benefits.

## 1. Introduction

The human intestine is colonized by a great number of microorganisms that contribute to host nutrition, metabolism, and immunity [1]. A structurally disrupted gut microbiota has been linked to the onset and development of various chronic diseases [2]. Dietary intervention to modulate the gut microbiota has become a potentially effective strategy to improve host health [3].

The concept of prebiotics was first introduced by Gibson and Roberfroid in 1995 [4], and over the years it has been updated a number of times to accommodate current knowledge [5]. The most recent version refers to “a substrate that is selectively utilized by host microorganisms conferring a health benefit” [6]. Previously, the “selective utilization” mostly referred to *Bifidobacterium* and *Lactobacillus*, whereas it is recognized today that prebiotic effects on the microbiota probably extend beyond these species. Fructans (fructo-oligosaccharides (FOS) and inulin) and galactans (galacto-oligosaccharides (GOS)) have been considered to be typical prebiotics [6]. *In vitro* studies and randomized controlled trials have shown that they stimulate the growth of *Bifidobacterium* populations [7,8], as well as certain butyrate-producing species [9,10]. In addition, numerous randomized controlled trials have demonstrated direct health benefits from GOS, FOS, and inulin, including the inhibition of pathogens, protective effects against cardiovascular disease, and the improvement of mineral bioavailability [11,12,13]. The increased production of short-chain fatty acids (SCFAs), following fermentation of prebiotics, is proposed to play a key role in their action mechanisms [14]. However, variations in prebiotic chemical structure, such as linkage type and the degree of polymerization, are known to affect their utilization by the gut microbiota, and thus SCFA output [15,16,17]. For GOS, most studies have been performed with the beta-linked form, whereas only a few studies exist on the prebiotic potential of alpha-linked GOS, and include a study that reported that the modulation of the microbiota composition is associated with beneficial effects on appetite [18,19,20].

Xylo-oligosaccharides (XOS) and beta-glucans are considered to be novel prebiotics [21,22] because, in contrast to inulin, FOS, and GOS, fewer studies have been performed to measure the health benefits related to the selective fermentation of XOS or beta-glucan by the host microbiota. XOS are sugar oligomers made up of xylose units, which are linked through beta-(1–4) linkages (Vazquez, 2000). In human studies, XOS consumption has been found to result in increased fecal *Bifidobacterium* populations, increased fecal concentrations of SCFAs, and reduced constipation in pregnant women [23,24]. Moreover, XOS supplementation was found to have effects on markers of immune function in healthy adults [25]. Studies with XOS also indicate the potential to improve the management of blood sugars and cholesterol [26,27]. Beta-glucan is a long-chain, soluble, viscous fiber that has physiological health benefits for cholesterol and glycemic control [28]. The prebiotic activity of beta-glucan from oat was demonstrated in rats [29] and more recently in an *in vitro* study [18].

In the present study, we investigated the fermentation profiles, including the production of SCFAs, of different dietary fibers with a range of concentrations (inulin, alpha-GOS, beta-GOS, XOS from corn cobs and high-fiber sugar cane, and beta-glucan), in comparison with a positive control (FOS) and a negative control (no fiber addition), using the intestinal microbiota fermentation screening (i-screen) platform.

## 2. Results

### 2.1. Effect of Fibers on Microbiota Composition

#### 2.1.1. Quantification of *Bifidobacterium* spp. and *Lactobacillus* Groups by Quantitative PCR (qPCR)

**Figure 1:**
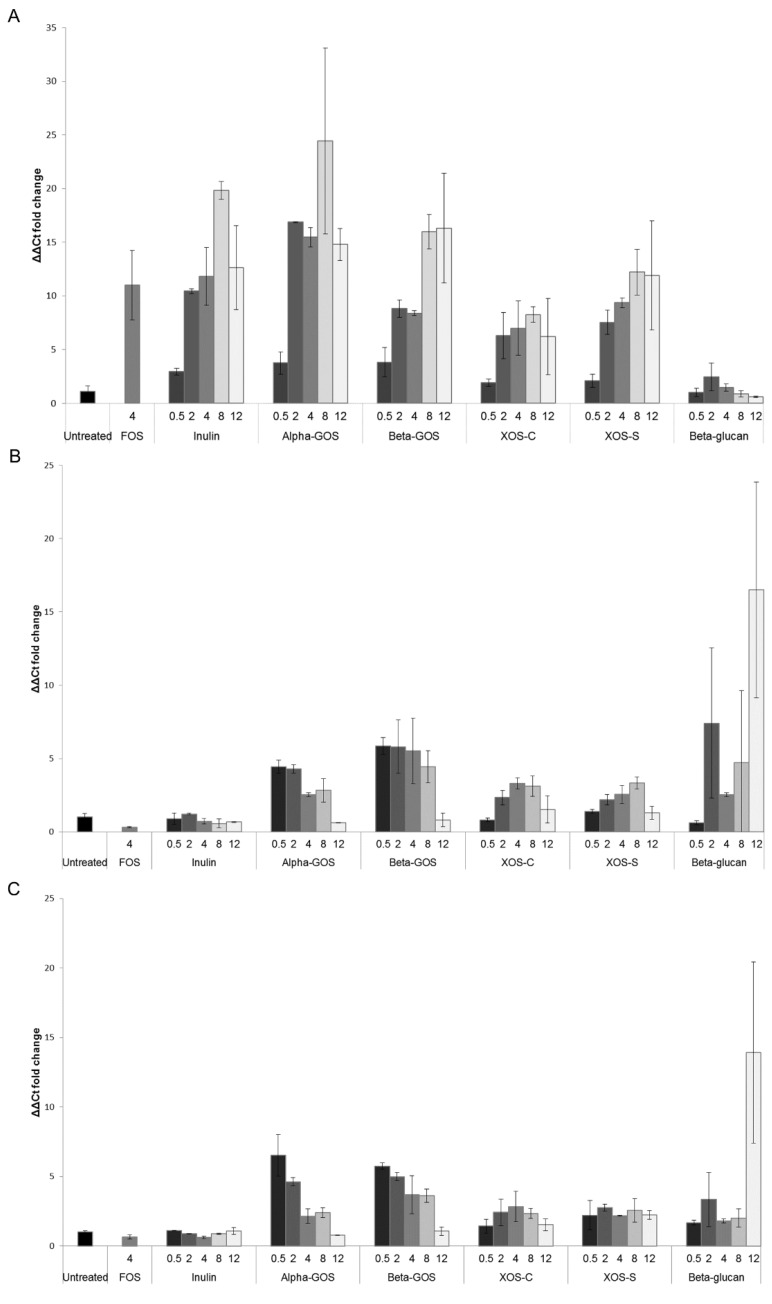
Mean relative fold change of bacterial groups in 24 h fermentation samples, as measured by qPCR. Values are expressed as the mean ± standard deviation, obtained from the average of duplicate (inulin, alpha-linked galacto-oligosaccharides (alpha-GOS), beta-linked galacto-oligosaccharides (beta-GOS), xylo-oligosaccharides from corn cobs (XOS-C), xylo-oligosaccharides from high-fiber sugar cane (XOS-S), and beta-glucan) or triplicate (control and fructo-oligosaccharides (FOS)) samples. (**A**) *Bifidobacterium*, (**B**) *Lactobacillus* group 1, and (**C**) *Lactobacillus* group 2.

The effect of the different fibers on *Bifidobacterium* spp. and *Lactobacillus* populations was assessed after 24 h of fermentation using qPCR (Figure 1). For all tested fibers, except beta-glucan, an increase in *Bifidobacterium* spp. was observed after 24 h of fermentation (Figure 1A). Alpha-GOS appeared to increase *Bifidobacterium* spp. to a greater extent than FOS, at a concentration of 4 mg/mL. Moreover, the increase in *Bifidobacterium* spp. seemed to occur in a dose-dependent manner for all fibers, except beta-glucan. Alpha-GOS also resulted in an increase in *Bifidobacterium* spp. in the order of approximately 15-fold, compared to an eight-fold increase for beta-GOS, at concentrations of 2 and 4 mg/mL. Inulin and FOS had no effect on *Lactobacillus* groups 1 and 2, whereas there was an increase in these populations for all other fibers (Figure 1B,C). These population increases were moderate, with the exception of beta-glucan at a concentration of 12 mg/mL, which stood out despite a large standard deviation. The changes observed in *Lactobacillus* groups 1 and 2 for alpha-GOS versus beta-GOS, as well as for xylo-oligosaccharides from corn cobs (XOS-C) versus XOS from high-fiber sugar cane (XOS-S), were comparable.

#### 2.1.2. Microbiota Profile Determined by 16S rRNA Gene Amplicon Sequencing

**Figure 2:**
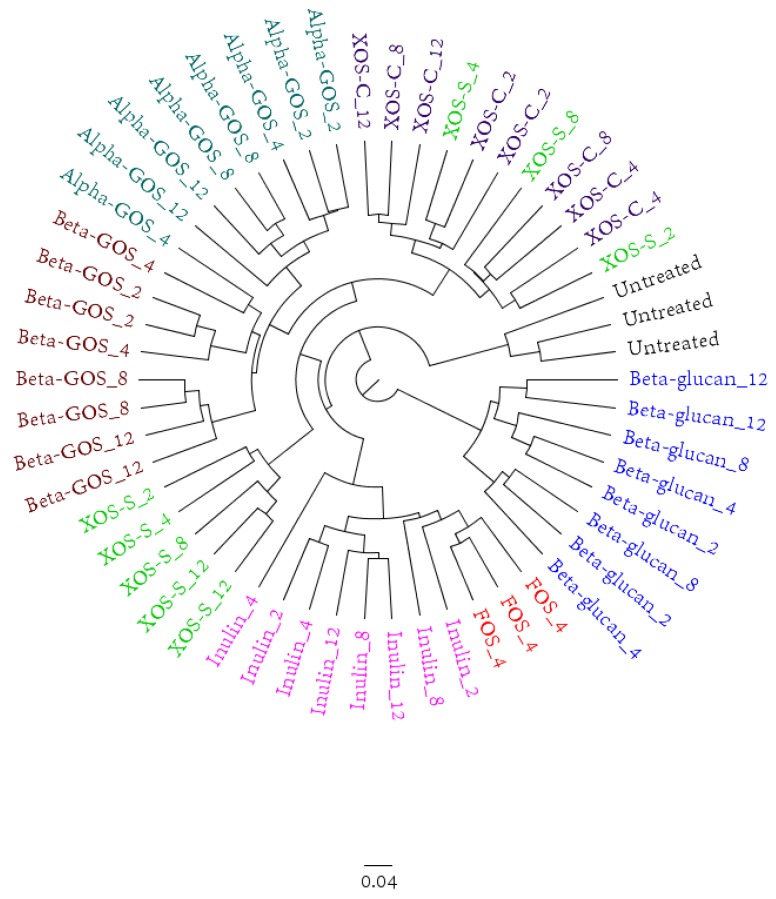
Clustering tree based on the Bray-Curtis dissimilarity of microbiota composition data, assessed by 16S rRNA gene amplicon sequencing. The number after the fiber indicates the concentration (in mg/mL) at which the fiber was added to the fermentation medium. The fiber concentration of 0.5 mg/mL is not depicted.

An overall representation of the results for the microbiota is given in Figure 2, which shows a clustering tree, based on a Bray–Curtis dissimilarity comparison, of all the data, except the 0.5 mg/mL fiber concentration that only had minor effects. The analysis illustrated that the effect of FOS on the microbiota was similar to inulin, as these samples clustered close to each other. Alpha-GOS-enriched samples showed changes similar to beta-GOS. For XOS-S, the duplicates appeared in two separate branches of the clustering tree, which indicated a large variance in the effects of this fiber on the microbiota. The beta-glucan samples clustered together and corresponded to an independent branch in the clustering tree, which indicated distinct changes in the composition of the microbiota compared to the other fibers.

The impact of the individual fiber substrates on the microbial taxa is displayed at the phylum and genus levels (Figures S1 and S2). With a relative abundance of 65%, Firmicutes was the dominant phylum in the untreated control, whereas Bacteroidetes and Actinobacteria accounted for 15% and 13%, respectively (Figure S1). The positive FOS control displayed a three-fold increase in Actinobacteria abundance at the expense of a 15% reduction in Firmicutes abundance, compared to the untreated control. This trend was observed across all fibers and appeared to be dose-dependent. Furthermore, there appeared to be a dose-dependent decrease in the relative abundance of Proteobacteria across all fibers. Genus-level profiles displayed a high and stable relative abundance of *Bacteroides* in the beta-glucan samples (Figure S2). This was also the case for XOS-C, whereas for all other fibers the abundance of *Bacteroides* decreased in a concentration-dependent matter.

**Figure 3:**
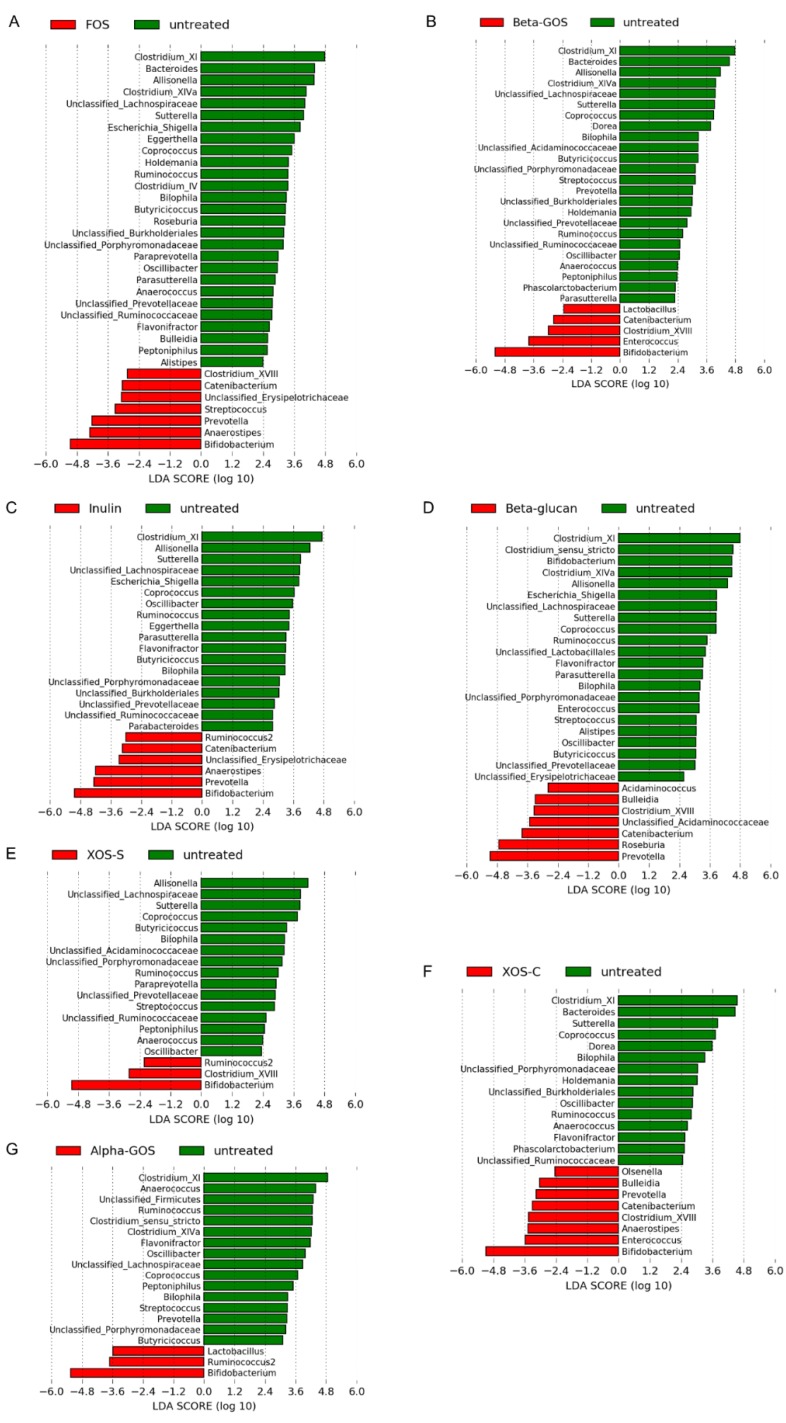
Identification of the most differentially abundant genera, between untreated controls and the fiber samples, using Linear Discriminant Analysis Effect Size (LEfSe) analysis. Genera enriched in the untreated samples are indicated with a positive Linear Discriminant Analysis (LDA) score (green), and genera enriched in the samples treated with different fibers are indicated with a negative LDA score (red). The threshold on the logarithmic LDA score for a discriminative genus was set to >2.0. (**A**) FOS, (**B**) Beta-GOS, (**C**) Inulin, (**D**) Beta-glucan, (**E**) XOS-S, (**F**) XOS-C, and (**G**) Alpha-GOS.

Linear Discriminant Analysis (LDA), or LDA Effect Size (LEfSe) analysis, was applied to sequences at the genus level in order to identify changes in the microbial composition between untreated controls and the different fibers (Figure 3). Across all fibers, except beta-glucan, *Bifidobacterium* exhibited the largest LDA score, which indicated that it was the most enriched genus compared to the untreated control. For beta-glucan, *Prevotella* and *Roseburia* were the most enriched genera and displayed the largest LDA scores. *Clostridium* cluster XI was the most distinguishing genus in the untreated control sample compared to all the fiber-treated samples, except for XOS-S of which *Allisonella* was the largest distinguishing genus.

The Shannon index, which accounts for species abundance and evenness, was calculated to assess the alpha-diversity of the samples (Table S1). All individual fiber substrates showed a decrease in alpha-diversity with increasing fiber concentrations.

### 2.2. Impact of Fibers on Metabolites

**Figure 4:**
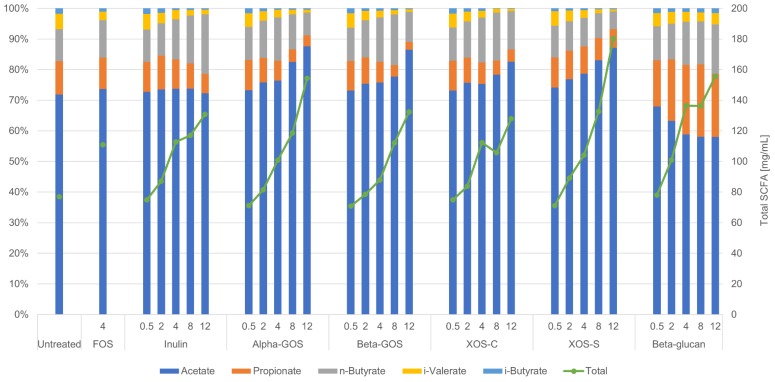
Total short-chain fatty acid (SCFA) concentrations and the relative percentages of SCFAs in the control and fiber-enriched samples. Acetate, propionate, butyrate, isovalerate, and isobutyrate were detected using gas chromatography (GC), and the total SCFA concentrations represent all metabolites measured.

The SCFAs acetate, propionate, butyrate, isovalerate, and isobutyrate could be detected in all fermentation samples (Figure 4). The individual SCFA production measurements can be seen in Table S2 Across all fibers, the total concentrations of SCFAs consistently increased with increasing fiber concentration. It is noteworthy that at 12 mg/mL of XOS-S, the total concentration of SCFAs surpassed that observed for all other fibers; a concentration of 180.3 mg/mL of SCFAs was observed with XOS-S compared to 127.4 mg/mL with XOS-C. The relative percentage patterns of the metabolites were comparable among the different fibers, except for beta-glucan which showed a distinct increase in the percentage of propionate at the expense of acetate.

There appeared to be a dose-response effect for butyrate with both inulin and beta-glucan fibers. The increase in butyrate formation, from the lowest to the highest fiber concentrations, was 7.9 to 25.5 mg/mL and 8.7 to 25.1 mg/mL for inulin and beta-glucan, respectively (Table S3).

The branched-chain fatty acid (BCFA) concentrations were low in all samples (0.91–5.77 mg/mL and 0.16–2.41 mg/mL for isovalerate and isobutyrate, respectively). The relative percentages of isobutyrate and isovalerate decreased in a concentration-dependent manner for all fibers, with the exception of beta-glucan for which the ratios remained essentially unchanged.

### 2.3. Dependence between the Microbiota and Metabolite Production

**Figure 5:**
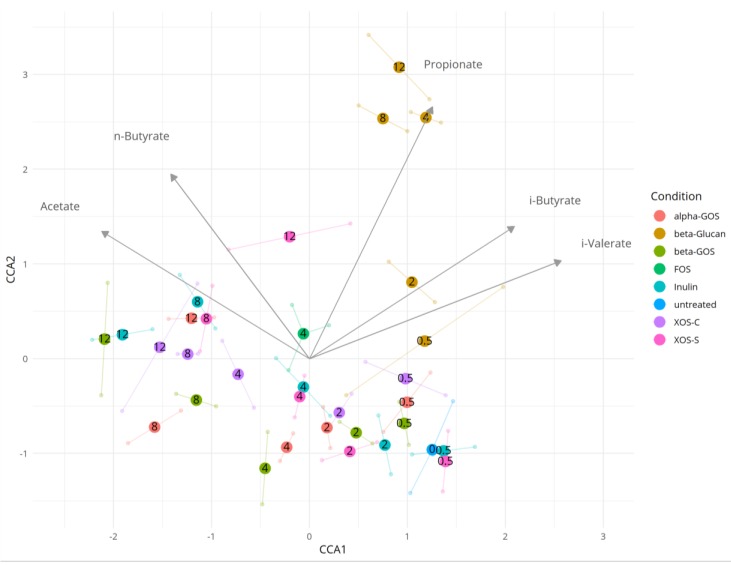
Relationship between the composition of the microbiota and the levels of short-chain fatty acids (SCFAs), using correspondence analysis. The larger dots represent microbiome composition and show the centroid values for each of the sets of duplicate samples. Arrows represent the direction of the association of microbiome composition with specific SCFA levels.

We visualized the relationship between the composition of the microbiota and the levels of SCFAs using canonical correspondence analysis (CCA) (Figure 5). Previous observations of the distinct effect of beta-glucan on the microbial composition were hereby reaffirmed in the CCA, where the samples fermented with beta-glucan at concentrations of 4, 8 and 12 mg/mL were segregated from all other samples. This appeared to be explained by the differences observed in the propionate levels for beta-glucan compared to the other fibers.

## 3. Discussion

The aim of the present study was to compare the effects of various dietary fiber types and concentrations on the microbial ecology of the human gut, using the i-screen platform to represent the large intestine. Previously, the i-screen model was validated with a concentration of 4.2 mg/mL of prebiotic [30]. Here, we investigated fibers in a concentration range of 0.5 to 12 mg/mL to capture dosage-dependent effects.

We found that all of the investigated fibers showed prebiotic effects, in terms of selective utilization by the microbiota. A bifidogenic effect was measured, with qPCR and gene amplicon sequencing, for all fibers except beta-glucan. At the phylum level, this effect emerged as an increase in the abundance of Actinobacteria. This is in line with several *in vivo* and *in vitro* studies that demonstrated bifidogenic effects of fructans and galactans [8,10,31,32,33], including alpha-GOS [34]. In addition, similar effects were also shown for XOS [35,36]. An increase in bifidobacteria can be regarded as a marker of intestinal health, with several studies demonstrating beneficial effects of this species on colorectal cancer, colon regularity, and acute diarrhea [37,38]. We observed no clear selective fermentation effect of *Bifidobacterium* in samples fermented with beta-glucan, which is consistent with other *in vitro* studies [39,40,41].

An enrichment of *Lactobacillus* spp. has been reported in some cases for GOS and FOS [42,43]. Our data showed an increase in lactobacilli mainly for beta-glucan, which has also been previously reported in the fecal microbiota of rats supplemented with beta-glucan [44], as well as in batch fermentation experiments [41]. However, there are also reports of beta-glucan having no effect on *Lactobacillus* spp. [39,40], suggesting that the effect might be dose-dependent because in our study a significant increase was observed only at the highest concentration.

The phylum Bacteroidetes and the genera *Prevotella* (members of Bacteroidetes) and *Roseburia* (members of Firmicutes) were most enhanced in response to beta-glucan. Both genera are abundant in the human gut [45,46]. *Prevotella* is associated with a high-fiber diet [47,48], and an increased abundance of *Roseburia* was observed in human volunteers on a diet high in resistant starch [49]. The increase in *Prevotella* in response to beta-glucan treatment is in agreement with a previous *in vitro* fermentation study [40], and may be partly explained by the presence of genes that are responsible for endo-beta-glucanase production, an enzyme essential for the digestion of beta-glucans [50,51]. A positive contribution of *Prevotella* to glucose tolerance was previously displayed in mice fed a high-fiber diet [48]. Notably, in our study, the increase in *Prevotella* with increasing concentrations of beta-glucan did not negatively affect the stable abundance of *Bacteroides*. This is in contrast to the often-observed inverse correlation of the two genera [52].

Microbial SCFAs have been shown to contribute significantly to host health within the gut and in the periphery [53]. Here, we found a dose-dependent increase in total SCFA concentrations for all fibers. Both forms of GOS and XOS induced an increased relative ratio of acetate. Acetate production pathways are widely distributed among bacterial groups in the gut [53]. It has been suggested that acetate has a direct role in central appetite regulation [54]. Additionally, acetate is known to undergo bacterial transformation into other metabolites, including butyrate, by so-called cross-feeding processes [55]. In the beta-glucan samples, we measured a higher propionate production compared to all other fibers, which may be explained by the promotion of *Prevotella*, a genus that contains important propionate-producing species, and *Roseburia*, which is one of the few genera that produces both butyrate and propionate [56]. Propionate has potential health-promoting effects due to its anti-lipogenic, cholesterol-lowering, anti-inflammatory, and anti-carcinogenic actions [57]. Furthermore, this SCFA may also play a role in appetite regulation [58].

Notably, the butyrate ratio increased with increasing concentrations of beta-glucan and inulin. Butyrate is an important energy source for intestinal epithelial cells and is believed to counteract colorectal cancer and inflammation [59]. This may be linked to the observed stimulation of the butyrate-producing species *Ruminococcus* and *Roseburia* by inulin and beta-glucan, respectively.

It was previously shown that variations in the chemical structure of a prebiotic can impact its selective fermentation by bacteria [60,61,62]. Here, we compared both GOS and XOS from different sources. The fermentation of beta-GOS and alpha-GOS resulted in comparable microbial and metabolic profiles. XOS of different origins (high-fiber sugar cane or corn cobs) also displayed similar effects on the composition of the microbiota and SCFA production, but some differences were observed at high concentrations, potentially due to the variations in purity as has been previously pointed out [62]. 

Clinical studies investigating prebiotic effects have some disadvantages with respect to ethical constraints, as well as limited sampling possibilities from the colon and limited measurements of *in situ* SCFA production, but these constraints are eliminated by applying an *in vitro* approach. However, the batch fermentation used in this study also has some limitations. The standardized fecal inoculum was pre-cultured at a pH of 5.8, which may have impacted the initial microbial composition of the fecal sample. However, it was previously reported that the compositional and metabolic changes in response to pre-culturing are insignificant [63]. Moreover, the stimulation of selected bacterial species, and the subsequent increase in metabolite production, led to a reduction in pH and thus a growth inhibition of some species. It is likely that the lack of pH control was the reason for the dose-dependent decrease in alpha-diversity that we observed for all tested fibers, which is a general phenomenon for batch fermentation experiments. An additional limitation was the short fermentation time of 24 h that failed to capture the complete picture of cross-feeding interactions between gut microbes, and which may not fully correlate with the long-term effects of fibers on the microbiota.

In conclusion, this study revealed changes in the adult fecal microbial ecology upon fermentation with different fibers. Many of the observed compositional and metabolic changes for typical prebiotics were in accordance with previous *in vitro* and *in vivo* data, thus confirming the suitability of the i-screen fermentation platform for the screening of novel prebiotic compounds. Our fermentation results support the prebiotic activity of alpha-GOS, XOS, and beta-glucan. The effects of XOS and alpha-GOS on the microbiota and metabolite production can be considered “prebiotic” in the classical sense of their ability to increase bifidobacterial populations. Beta-glucan, on the other hand, induced distinct changes compared to well-established prebiotics. The observed increases in butyrate and propionate may be linked to the health benefits of beta-glucan. Further *in vivo* human studies may help to strengthen the link between the beneficial health effects of beta-glucan, notably on glucose metabolism, and the changes induced in the gut microbiota.

## 4. Materials and Methods

### 4.1. Fibers

The characteristics of the fibers (FOS, inulin, alpha-GOS, beta-GOS, XOS-C, XOS-S and beta-glucan) investigated in the i-screen fermentation platform are described in Table 1.

### 4.2. Fecal Inoculum

As an inoculum for the i-screen platform, a standardized human adult intestinal microbiota sample was used. Fecal samples were collected from six healthy adult volunteers (Caucasian individuals, subject to a European lifestyle and nutrition, and no antibiotic usage in the last three months) in a closed box with an anaerobic strip (AnaeroGen, Oxoid, Cambridge, UK) inside, as described previously [63]. To create the standardized microbiota, pooled stools were grown in a fed-batch fermenter for 40 h. The fermentation medium was based on the standard ileal efflux medium (SIEM) composition [64] that was modified as described previously [63] and adjusted to a pH of 5.8. This standard adult gut microbiota was stored at −80 °C in 12% glycerol. Collection of fecal samples was performed anonymously following TNO standard operational procedures, which was approved by an internal ethical evaluation board and is in compliance with the Dutch laws on medical/scientific research. Participants gave written informed consent.

### 4.3. Experimental Set-Up

Before starting the i-screen incubations with the test materials, the standardized fecal inoculum was incubated in the modified SIEM overnight (37 °C; 300 rpm) using a Whitley A45 anaerobic cabinet (Kentron Microbiology BV, Doetinchem, the Netherlands) and an 80% N_2_/10% CO_2_/10% H_2_ gas mixture to activate the fecal bacteria. Then, the fibers were mixed with SIEM and the 1% (*v*/*v*) fecal inoculum in each well of a deep-well plate. Five concentrations (0.5, 2, 4, 8, and 12 mg/mL) of inulin, alpha-GOS, beta-GOS, XOS-C, XOS-S, and beta-glucan fibers were tested in duplicate. Due to viscosity issues, the two highest concentrations of beta-glucan (8 and 12 mg/mL) had to be weighed into the test wells directly and were not dissolved beforehand. Inoculated SIEM without fiber was used as a negative control, and supplementation with FOS at 4 mg/mL was used as a positive prebiotic control. Both controls were included in triplicate. After 24 h of fermentation, collected samples were directly stored at −20 °C for subsequent DNA isolation and SCFA analysis.

### 4.4. DNA Isolation

Total DNA from collected samples was isolated as described by Ladirat et al. [63] with some minor adjustments: The samples were initially mixed with 300 μL of lysis buffer (Agowa, Berlin, Germany), 500 μL of zirconium beads (0.1 mm), and 500 μL of phenol, before being placed in a BeadBeater (BioSpec Products, Bartlesville, OK, USA) for 3 min.

### 4.5. Quantitative PCR (qPCR)

Total DNA was used for qPCR analyses using TaqMan chemistry. The primers and probes used to quantify the total number of bacteria in *Lactobacillus* group 1 (which includes *L. gasseri*, *L. helveticus*, *L. johnsonii*, *L. amylovorus*, *L. acidophilus*, *L. delbrueckii*, *L. crispatus*, *L. jensenii*, *L. amylolyticus*, and *L. kefiranofaciens*), *Lactobacillus* group 2 (which includes *L. casei*, *L. plantarum*, *L. brevis*, *L. salivarius*, *L. paracasei*, *L. reuteri*, *L. rhamnosus*, *L. buchneri*, *L. fermentum*, *L. pentosus*, *L. animalis*, *L. sakei*, and *L. murinus*), and *Bifidobacterium* spp. are described in Table S3.

qPCR was performed on an Applied Biosystems 7500 thermal cycler, using the TaqMan^®^ Fast Universal PCR Master Mix. For the real-time PCR, 5 μL of DNA, 12.5 μL of TaqMan^®^ Fast Universal PCR Master Mix, 1 μL (10 pmol) of forward and reverse primers, and 1 μL (5 pmol) of TaqMan probe were mixed, and 4.5 μL of DNase-free water was added, to make a final volume of 25 μL. The cycling conditions consisted of 3 min at 95 °C, followed by 45 cycles of 3 s at 95 °C, and then 30 s at 60 °C. Real-time data were analyzed with Applied Biosystems 7500 software (Version 1.4). Upon completion of the run, a cycle threshold (*C*t) was calculated. Relative fold change values were obtained by the ∆∆*C*t method, where all results are normalized to the 16S data (∆*C*t) and the untreated control samples are utilized as the control (∆∆*C*t).

### 4.6. 16S rRNA Gene Amplicon Sequencing and Analysis

To determine changes in the composition of the microbiota of fermentation samples with individual fiber substrates, 16S rRNA gene amplicon sequencing was performed. A total of 100 pg of DNA was amplified, targeting the V4 hypervariable region, using the F515/R806 primers as described previously [65], with the exception that 30 cycles were used instead of 35 [66]. Primers included Illumina adapter sequences and a unique 8-nt sample index sequence key [65]. To determine the amount of bacterial DNA, qPCR, using primers specific for the bacterial 16S rRNA gene, was carried out. The amplicon libraries were pooled in equimolar amounts and purified using the QIAquick Gel Extraction Kit (QIAGEN, Hilden, Germany). Amplicon quality and size were analyzed on a Fragment Analyzer (Advanced Analytical Technologies, Inc., Heidelberg, Germany). Paired-end sequencing of amplicons were conducted on the Illumina MiSeq platform (Illumina, Eindhoven, The Netherlands).

Pre-processing, analysis, and classifications of sequencing data were performed using modules implemented in the Mothur software platform [67]. Chimeric sequences were identified and removed using the chimera.uchime command. Unique 16S rRNA sequences were aligned using the align.seqs command and the Mothur-compatible Bacterial SILVA SEED database (Release 119; available online: https://mothur.org/wiki/Silva_reference_files). Bacterial sequences were taxonomically classified by the RDP-II Naïve Bayesian Classifier using a 60% confidence threshold against the RDP Database (Release 11.1; available online: https://www.mothur.org/wiki/RDP_reference_files) for 16S rRNA. Richness and diversity including the Shannon diversity index were calculated.

### 4.7. Gas Chromatography Analysis

SCFAs (acetate, propionate, and n-butyrate) and BCFAs (isobutyrate and isovalerate) were analyzed as described by Jouany [68], and modified slightly as described by Van Nuenen [69]. In brief, exposed material from the i-screen samples was centrifuged (~12,000× *g*, 5 min) and the cleared supernatant was filter sterilized (0.45 µm). A mixture of formic acid (20%), methanol, and 2-ethyl butyric acid (internal standard, 2 mg/mL in methanol) was added. A 3 µL sample, with a split ratio of 75.0, was injected on a GC-column (ZB-5HT inferno, ID 0.52 mm, film thickness 0.10 µm; Zebron, Phenomenex, Torrance, CA, USA) in a Shimadzu GC-2014 gas chromatograph (Shimadzu Europa GmbH, Duisburg, Germany).

### 4.8. Statistical Analysis

All qPCR data are reported as the mean ± SD of duplicates which does not allow for statistical inference and hypothesis testing.

Statistical analysis of the 16S rRNA amplicon sequencing data was performed using LEfSe [70]. For the LEfSe analysis, the non-parametric factorial Kruskal-Wallis (KW) sum-rank test, with an alpha of 0.05, was used to detect genera with significant differential abundance of each fiber with respect to the control. The biological significance was subsequently investigated using a set of pairwise tests among the different fiber concentrations and the control, using the (unpaired) Wilcoxon rank-sum test with an alpha of 0.05. As a last step, LEfSe uses Linear Discriminant Analysis to estimate the effect size of each differentially abundant genus. The threshold on the logarithmic LDA score for a differentially abundant genus was set to >2.0.

16S rRNA gene amplicon sequencing data and environmental variables (SCFA levels) were ordinated using CCA as implemented in the R package “vegan” [71], in R version 3.4.4 [72]. Canonical correspondence analysis is a multivariate constrained ordination technique that extracts major gradients among combinations of explanatory variables. The same package was used to calculate the Shannon index of alpha-diversity. Distance based analyses (CCA and clustering) were performed using the Bray-Curtis dissimilarity measure.

### 4.9. Data Availability

All DNA sequences presented in this study will be deposited in the sequence read archive (SRA) of the NCBI database.

## Figures and Tables

**Table 1:** Characteristics of fibers used in the *in vitro* fermentation.

Fiber	Source	Purity	Supplier
FOS	Chicory root	≥90%	Sigma-Aldrich, Zwijndrecht, The Netherlands
Inulin	Chicory root	≥99.5%	SENSUS, Roosendaal, The Netherlands
Alpha-GOS	Peas	≥95%	SAS Olygose, Venette, France
Beta-GOS	Lactose	90%	Hylen Co., Qingdao, China
XOS-C	Corn cobs	≥95%	Longlive Biotechnology Co., Shandong, China
XOS-S	Sugar cane fiber	≥75%	Prenexus Health Inc., Gilbert, AZ, USA
Beta-glucan	Oat flour	>94%	Megazyme, Wicklow, Ireland

## Supplementary Materials

Supplementary materials can be found at http://www.mdpi.com/1422-0067/19/10/3097/s1.
