# Supplementary material for: In Vitro Fermentation of Selected Prebiotics and Their Effects on the Composition and Activity of the Adult Gut Microbiota
Source: Int J Mol Sci. 2018 Oct 10;19(10):3097. doi: 10.3390/ijms19103097 (PMC6213619; doi:10.3390/ijms19103097)
Supplement: Supplementary file 1 [file ijms-19-03097-s001.pdf]

Supplementary

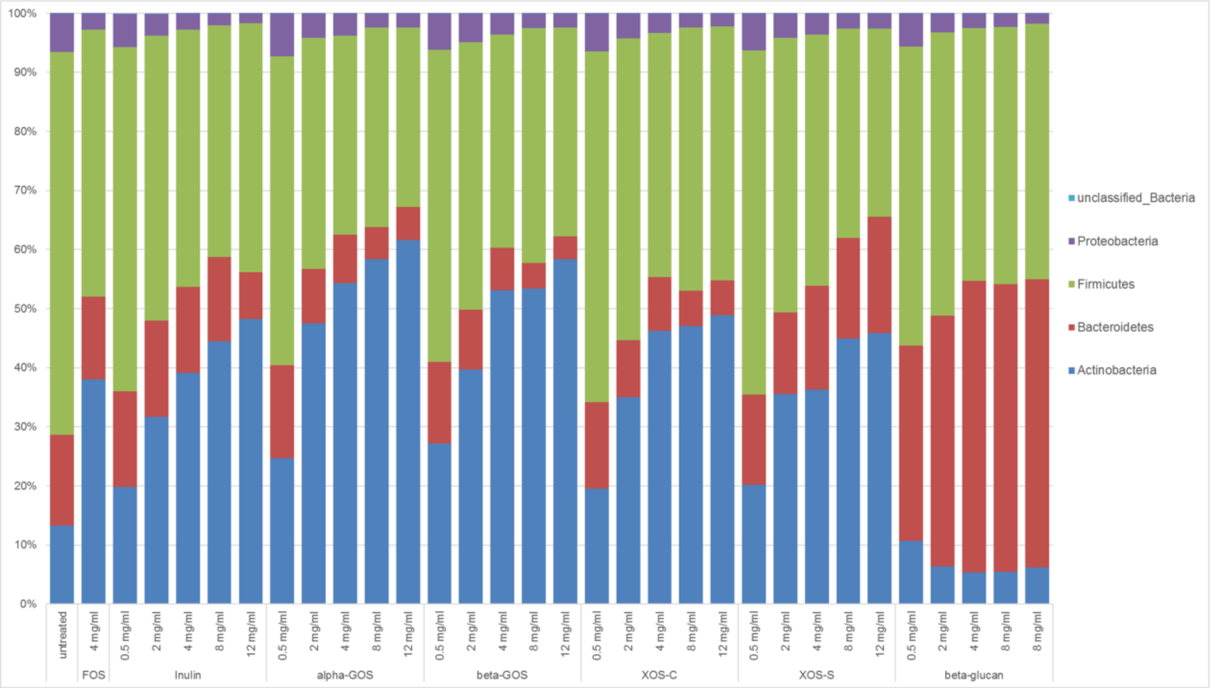

Figure S1. Sequencing Phylum.

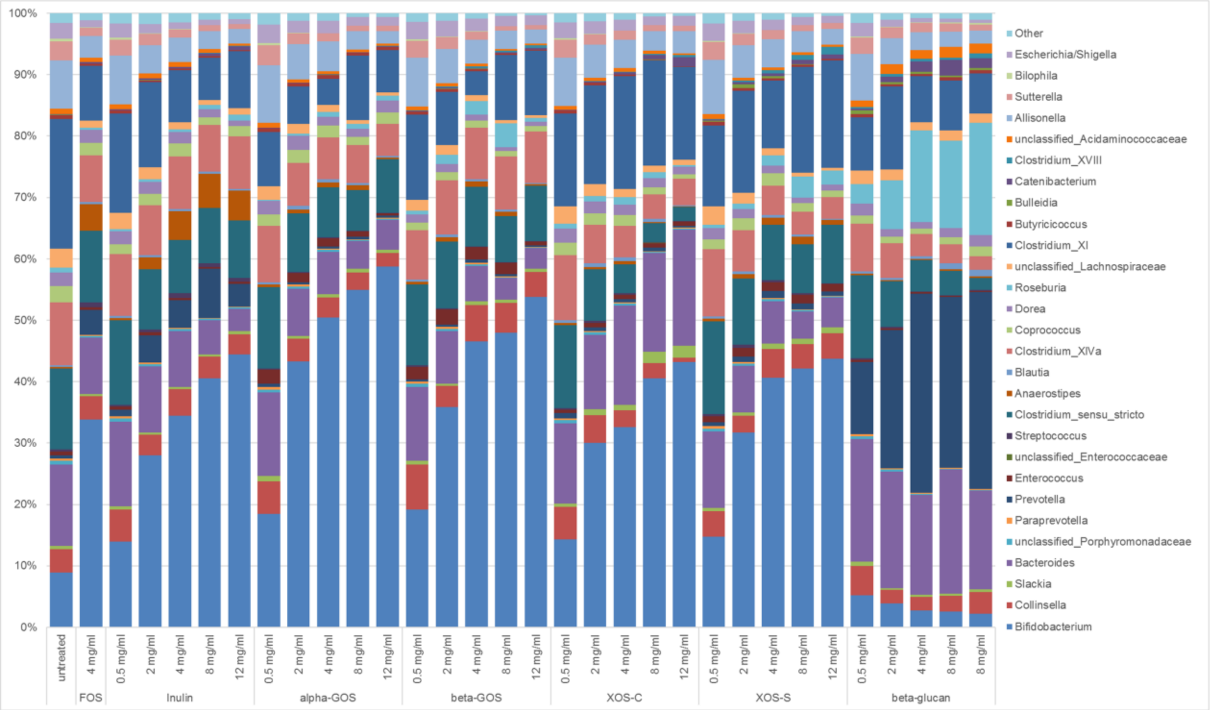

Figure S2. Sequencing Genus.

Table S1. Mean Shannon diversity index of all samples. Values are expressed as the mean ± standard deviation obtained from the average of duplicate (inulin, alpha-GOS, beta-GOS, XOS-C, XOS-S, beta-glucan) or triplicate (control and FOS) samples.

Condition	Concentration	Shannon index
Untreated		5.14 ± 0.42
FOS	4	5.22 ± 0.13
Inulin	0.5	5.27 ± 0.31
	2	4.72 ± 0.26
	4	4.91 ± 0.90
	8	4.40 ± 0.51
	12	4.18 ± 0.29
Alpha-GOS	0.5	5.85 ± 0.12
	2	5.10 ± 0.09
	4	4.84 ± 0.69
	8	4.43 ± 0.40
	12	4.13 ± 0.21
Beta-GOS	0.5	5.67 ± 0.05
	2	4.74 ± 0.34
	4	4.89 ± 0.60
	8	4.54 ± 0.01
	12	4.31 ± 0.01
XOS-C	0.5	5.25 ± 0.65
	2	4.72 ± 0.25
	4	5.20 ± 0.62
	8	4.72 ± 0.88
	12	4.81 ± 0.10
XOS-S	0.5	5.25 ± 0.60
	2	5.41 ± 0.21
	4	4.76 ± 0.23
	8	5.21 ± 0.26
	12	4.50 ± 0.23
Beta-glucan	0.5	5.53 ± 0.59
	2	4.76 ± 0.54
	4	4.44 ± 0.72
	8	5.51 ± 0.38
	12	4.93 ± 0.82

Table S2. Mean metabolite concentrations (mg/ml) of all samples. Values are expressed as the mean ± standard deviation obtained from the average of duplicate (inulin, alpha-GOS, beta-GOS, XOS-C, XOS-S, beta-glucan) or triplicate (control and FOS) sample.

Condition	Con-centration	Acetate	Propionate	n-Butyrate	i-Valerate	i-Butyrate
Untreated		5.14 ± 0.42	8.43 ± 0.76	8.03 ± 0.96	3.82 ± 0.57	1.39 ± 0.18
FOS	4	55.22 ± 5.34	11.41 ± 0.55	13.56 ± 0.08	3.09 ± 0.07	1.19 ± 0.07
Inulin	0.5	54.34 ± 2.95	7.34 ± 2.96	7.89 ± 0.51	3.78 ± 0.48	1.40 ± 0.04
	2	63.98 ± 1.26	9.61 ± 0.13	9.24 ± 0.67	3.00 ± 0.10	1.26 ± 0.03
	4	83.17 ± 2.74	10.84 ± 0.25	14.73 ± 0.75	3.50 ± 0.15	0.57 ± 0.20
	8	86.30 ± 1.07	9.58 ± 0.02	18.36 ± 1.66	2.06 ± 0.10	0.64 ± 0.09
	12	94.49 ± 0.53	8.29 ± 0.10	25.45 ± 0.28	1.94 ± 0.14	0.63 ± 0.04
Alpha-GOS	0.5	52.02 ± 0.66	7.03 ± 0.17	7.65 ± 0.05	3.19 ± 0.12	1.12 ± 0.04
	2	61.78 ± 2.88	6.48 ± 0.28	9.89 ± 0.65	2.52 ± 0.01	0.77 ± 0.12
	4	77.07 ± 5.19	6.50 ± 0.37	14.30 ± 1.03	2.56 ± 0.03	0.45 ± 0.01
	8	97.81 ± 3.15	4.94 ± 0.04	13.54 ± 0.59	1.74 ± 0.11	0.56 ± 0.16
	12	135.09 ± 1.11	5.56 ± 0.23	11.47 ± 2.25	1.50 ± 0.06	0.63 ± 0.10
Beta-GOS	0.5	51.78 ± 0.32	6.81 ± 0.50	7.73 ± 0.03	3.31 ± 0.10	1.15 ± 0.01
	2	59.12 ± 1.25	6.73 ± 0.09	9.51 ± 0.39	2.38 ± 0.06	0.68 ± 0.02
	4	66.52 ± 1.64	6.03 ± 0.16	12.71 ± 1.09	1.93 ± 0.04	0.66 ± 0.01
	8	87.13 ± 0.15	4.23 ± 1.28	18.56 ± 0.06	1.53 ± 0.05	0.68 ± 0.10
	12	114.41 ± 2.88	3.30 ± 1.38	13.06 ± 0.07	1.07 ± 0.05	0.40 ± 0.08
XOS-C	0.5	54.69 ± 2.92	7.25 ± 0.27	8.16 ± 0.58	3.41 ± 0.32	1.26 ± 0.25
	2	63.41 ± 0.48	6.91 ± 0.03	9.89 ± 038	2.70 ± 0.10	0.88 ± 0.09
	4	84.43 ± 1.35	7.95 ± 0.50	16.40 ± 1.33	2.51 ± 0.07	0.90 ± 0.08
	8	82.82 ± 6.82	4.83 ± 0.96	16.62 ± 0.85	1.26 ± 0.10	0.17 ± 0.05
	12	105.61 ± 0.20	5.18 ± 0.09	16.00 ± 3.95	0.91 ± 0.06	0.25 ± 0.08
XOS-S	0.5	52.73 ± 0.46	6.99 ± 0.60	7.41 ± 0.15	3.36 ± 0.14	0.66 ± 0.06
	2	68.50 ± 1.03	8.29 ± 0.26	8.58 ± 0.05	3.11 ± 0.08	0.62 ± 0.31
	4	81.90 ± 0.44	9.36 ± 0.31	9.71 ± 0.10	2.58 ± 0.03	0.62 ± 0.50
	8	110.11 ± 3.16	9.71 ± 0.13	10.68 ± 0.47	1.76 ± 0.05	0.35 ± 0.27
	12	157.10 ± 0.95	11.00 ± 0.43	10.48 ± 0.33	1.28 ± 0.05	0.45 ± 0.02
Beta-glucan	0.5	52.96 ± 0.59	11.75 ± 0.26	8.68 ± 0.08	3.39 ± 0.03	1.17 ± 0.00
	2	63.71 ± 1.17	20.27 ± 0.86	11.79 ± 0.61	3.85 ± 0.02	1.17 ± 0.14
	4	80.17 ± 2.10	31.04 ± 0.39	19.26 ± 2.20	4.42 ± 0.07	1.53 ± 0.04
	8	79.12 ± 2.31	32.22 ± 7.91	19.08 ± 2.56	4.13 ± 0.60	1.73 ± 0.18
	12	90.25 ± 0.79	32.17 ± 2.86	25.07 ± 2.69	5.77 ± 0.32	2.41 ± 0.29

Table S3. Primers and probes used for enumeration of bacterial groups by qPCR

Target	Forward primer	Reverse primer	Probe	Chemistry	Reference
Total bacteria (16S uni1)	5′ CGAAAGCGTG GGGAGCAAA 3′	5′ GTTCGTACTC CCCAGGCGG 3′	5′ ATTAGATACC CTGGTAGTCC A 3′	FAM/MGB	[1]
Lactobacillus group 1	23S-LBgr1-F CGCCGMAAG ACTAAGGTTT CCT	23S-LBgr1-R GCCTYRCCTT AGGTCCCG	23S-LBgr1-probe AGGCTCGTCC GCCCAG	FAM/MGB	TNO
Lactobacillus group 2	23S-LBgr2-F CCACCGWAT GACTAAGGTT TCCTG	23S-LBgr2-R GGCCTCGVCT TAGRTCCC	23S-LBgr2-probe AGGCTCGTCC TCCCAG	VIC/MGB	TNO
Bifidobacterium	IS-aIIBif-F GGGATGCTG GTGTGGAAGA GA	IS-aIIBif-R TGCTCGCGTC CACTATCCAG T	IS-aIIBif TCAAAC CACCACGCG CCA	FAM/MGB	[2]

TNO, The Netherlands Organization for Applied Scientific Research

References

1. de Oliveira, J.E.; van der Hoeven-Hangoor, E.; van de Linde, I.B.; Montijn, R.C.; van der Vossen, J.M. In ovo inoculation of chicken embryos with probiotic bacteria and its effect on posthatch Salmonella susceptibility. *Poult Sci* **2014**, *93*, 818-829.
2. Haarman, M.; Knol, J. Quantitative real-time PCR assays to identify and quantify fecal *Bifidobacterium* species in infants receiving a prebiotic infant formula. *Appl Environ Microbiol* **2005**, *71*, 2318-2324.
